# A Deep Learning-Based Method for Preventing Data Leakage in Electric Power Industrial Internet of Things Business Data Interactions

**DOI:** 10.3390/s24134069

**Published:** 2024-06-22

**Authors:** Weiwei Miao, Xinjian Zhao, Yinzhao Zhang, Shi Chen, Xiaochao Li, Qianmu Li

**Affiliations:** 1 State Grid Jiangsu Electric Power Co., Ltd., Information & Telecommunication Branch, Nanjing 210024, China; 2 School of Cyber Science and Engineering, Nanjing University of Science and Technology, Nanjing 210094, China; lixiaochao@njust.edu.cn (X.L.); qianmu@njust.edu.cn (Q.L.)

**Keywords:** industrial internet of things, data leakage prevention, blockchain, electric power data, pre-trained language model

## Abstract

In the development of the Power Industry Internet of Things, the security of data interaction has always been an important challenge. In the power-based blockchain Industrial Internet of Things, node data interaction involves a large amount of sensitive data. In the current anti-leakage strategy for power business data interaction, regular expressions are used to identify sensitive data for matching. This approach is only suitable for simple structured data. For the processing of unstructured data, there is a lack of practical matching strategies. Therefore, this paper proposes a deep learning-based anti-leakage method for power business data interaction, aiming to ensure the security of power business data interaction between the State Grid business platform and third-party platforms. This method combines named entity recognition technologies and comprehensively uses regular expressions and the DeBERTa (Decoding-enhanced BERT with disentangled attention)-BiLSTM (Bidirectional Long Short-Term Memory)-CRF (Conditional Random Field) model. This method is based on the DeBERTa (Decoding-enhanced BERT with disentangled attention) model for pre-training feature extraction. It extracts sequence context semantic features through the BiLSTM, and finally obtains the global optimal through the CRF layer tag sequence. Sensitive data matching is performed on interactive structured and unstructured data to identify privacy-sensitive information in the power business. The experimental results show that the F1 score of the proposed method in this paper for identifying sensitive data entities using the CLUENER 2020 dataset reaches 81.26%, which can effectively prevent the risk of power business data leakage and provide innovative solutions for the power industry to ensure data security.

## 1. Introduction

With the accelerated advancement of technologies like cloud computing, artificial intelligence, and blockchain, the Industrial Internet of Things has emerged as a pivotal force driving the digital, networked, and intelligent evolution of industries, serving as a catalyst for societal progress. Among its vital branches, the Electric Power Industrial Internet of Things stands out for its dedication to seamlessly integrating Internet technology into the power system, aiming to foster intelligent and efficient power production, transmission, distribution, and management [1]. The blockchain-based Industrial Internet of Things (BIIoT) ensures the security and transparency of data through its distributed ledger and non-tamperable characteristics, while improving the data management and collaboration efficiency of the power system [2,3].

In the realm of the Power Industrial Internet of Things, ensuring data security and privacy protection is paramount [4]. Given the vast amount of sensitive information inherent in the power system, including power supply network topology, user power data, equipment status, and more, any breach of these data could precipitate severe security vulnerabilities and substantial economic losses. The distributed storage of the blockchain ensures the decentralized storage of data in multiple nodes, further enhancing the security and reliability of data and preventing single points of failure and unauthorized access [5]. The types of participants in the new power system are gradually increasing, the market transaction mechanism is becoming increasingly complex, and data interactions are becoming more frequent. The collection and use of data are important driving forces for the sustainable development of smart grids in the era of artificial intelligence. The application of artificial intelligence technology has brought new changes to the development of the electric power industry and clarified the innovative development direction of electric power artificial intelligence.

### 1.1. Research Motivation

However, due to the particularity of the data in question, in the processes of open circulation and sharing, problems are faced such as difficulty in confirming the ownership of data assets, difficulty in traceability and anti-counterfeiting, difficulty in cross-domain mutual trust, and difficulty in security management. Data sharing has a certain risk of privacy leakage. The widespread advancement of deep learning has facilitated the development of private information sharing [6]. At the same time, private data are characterized by structural diversity. In this information age, information security and personal security are top priorities [7,8,9].

The State Grid business platform will interact with power business data with third-party platforms such as third-party energy management platforms, external Internet of Vehicles platforms [10], external source network load storage management systems, and comprehensive energy management platforms. For the electric power field, there are many data circulation paths, high demand for large-scale data processing, and diversified data types. The uploading and downloading of data may cause data leakage [11,12,13]. At the same time, the State Grid business platform needs to obtain data from the government and external enterprises, share data with the government and external enterprises, and conduct data security reviews. There is a demand for the security of data information interactions [14,15]. With the development of artificial intelligence technology, third-party platforms may be attacked to a certain extent, and data between the third-party platform and the State Grid business platform may be leaked.

With the excellent performance and strong generalization ability of deep learning methods in identifying and processing data, we propose a data interaction leakage prevention method based on deep learning that combines regular expressions with DeBERTa (Decoding-enhanced BERT with disentangled attention) and BiLSTM (Bidirectional Long Short-Term Memory)-CRF (Conditional Random Field). These models are combined to improve accuracy and generalization. Specifically, this method is based on the DeBERTa model, and uses the disentangled attention mechanism and enhanced mask decoder to perform pre-training feature extraction and provide input for the subsequent BiLSTM. Then, the forward and backward hidden states are considered at each time step through the BiLSTM, and the sequence context semantic features are extracted. Finally, the globally optimal label sequence is obtained through the CRF layer. Sensitive data matching is performed on structured and unstructured data to identify privacy-sensitive information in power business interactive.

### 1.2. Research Contributions

In summary, the main contributions of this article are as follows:

1. This article proposes a deep learning-based anti-leakage method for power business data interactions that can ensure the security of power business data interactions between the State Grid business platform and third-party platforms, thus fortifying the overall data security of the Power Industry Internet of Things (IoT).
2. The DeBERTa and BiLSTM-CRF models are combined. The DeBERTa model is improved based on the BERT model, combining the decoupled attention mechanism and enhanced mask decoder. It not only considers the relative position of the context, but also introduces absolute position information, which can improve forecasting capabilities. BiLSTM can capture contextual information in a sequence, thereby effectively improving the matching effect of sensitive data in the power business.
3. Experiments were conducted on the CLUENER 2020 dataset. The experimental results show that the method proposed in this article can effectively identify sensitive power business data, prevent the risk of power business data leakage, and provide reliable guarantee for the security of power business data interactions, thereby offering an innovative solution for enhancing data security in the Power Industry Internet of Things.

### 1.3. Article Organization

The remainder of this paper is structured as follows: Section 2 provides a brief overview of related work on data leakage prevention and the BERT model. Section 3 delves into the details of the proposed anti-leakage method for power business data interaction, which is based on regular expressions and the BERT model. In Section 4, experiments are conducted to showcase the feasibility and advantages of the method. Finally, Section 5 summarizes the outcomes of our work, along with future research topics and directions.

## 2. Related Work

### 2.1. Data Leakage Prevention

Data leakage occurs when an individual’s or organization’s private, confidential information is released into an insecure environment, whether intentionally or unintentionally [16]. With the proliferation of database services on the Internet, data passing through unstable networks may become vulnerable [17,18]. Leaked information often includes sensitive data on employees and customers, some of which may be unusable for malicious purposes [19]. Consequently, data leakage significantly impacts a company’s reputation. To mitigate these risks, various data leakage prevention technologies have emerged.

The primary distinction between data leakage prevention technology and traditional security controls like firewalls, VPNs, and IDS lies in their focus and approach [20]. Traditional security controls typically prioritize network perimeter defense and pay less attention to the actual content of the data. Mogull et al. [21] observed that context-based data leakage prevention systems concentrate on the contextual information surrounding sensitive data to identify potential leaks.

Content-based analytics data leakage prevention systems are more common and preferable than context-based analytics data leakage prevention systems because it makes more sense to prioritize safeguarding the data itself rather than concentrating solely on the surrounding circumstances. Sensitive data in a repository or in transit are typically monitored by content-based data leakage prevention systems, which mainly use statistical analysis, data fingerprinting, and regular expressions. Regular expressions are frequently employed in conjunction with specific rules, like identifying phone numbers and identity card numbers. The problem with data breach prevention systems that use regular expression analysis is their limited data protection and substantial rate of false positives [22].

Data breach prevention systems employing data fingerprinting can offer an enhanced coverage of sensitive data by detecting and preventing breaches of entire documents or segments thereof. Nonetheless, conventional fingerprinting may lose track when sensitive data undergo alterations or modifications. This occurs due to the susceptibility of traditional hashes, such as MD5 and SHA1, to changes [23]. Even minor adjustments in data can yield entirely distinct fingerprints upon hashing.

Kantor et al. [24] found that this may cause data to bypass the data leakage prevention system, thus leading to data leakage. To address this issue partially, multiple data hashing can be employed. In this approach, the initial data are segmented into smaller units, such sentences and paragraphs, and each unit is hashed separately. This guarantees that a certain amount of the initial fingerprint data can still be retrieved. These smaller fingerprints are more susceptible to alteration though, and even small adjustments can make the technique useless. More sophisticated methods try to address this challenge by employing similarity summaries [25], implementing Rabin fingerprinting [26], or utilizing piecewise hashing [27]. Nonetheless, these solutions have limited efficacy and may encounter various text obfuscations.

Blockchain technology provides a strong guarantee for data leakage prevention through its distributed ledger, non-tamperability, encrypted communication, smart contracts, transparency, and decentralization features. These features are particularly important in sensitive fields such as the Power Industry Internet of Things, and effectively prevent data leakage and ensure data security and integrity.

### 2.2. Pre-Trained Language Model

At the end of 2018, Google proposed a new language model called BERT (Bidirectional Encoder Representations from Transformers) [28]. The model is engineered to conduct deep learning on bidirectional text, facilitating its integration into machine learning frameworks. Its key advantage lies in its simplicity and simplicity of usage, simply requiring an output layer to be added to an already-existing neural network design. This enables the creation of a text model that surpasses the accuracy of all existing models across various natural text processing tasks. Robustly optimized BERT approach (RoBERTa) [29] is a pre-trained language representation model based on BERT improvements. RoBERTa adopts a more flexible masking strategy by masking parts of the words in the input text and requiring the model to predict the positions of these masks, instead of randomly masking 50% of the words like BERT. By eliminating the Next Sentence Prediction (NSP) job from BERT, RoBERTa concentrates on the single-sentence masked language model (MLM) challenge. In addition, Yang et al. [30] proposed the XLNet model, which learns bidirectional context by maximizing the expected likelihood of all permutations of the decomposition sequence, and incorporates the idea of the advanced autoregressive model Transformer-XL into pre-training. The application of the BERT model in various NLP tasks has also been widely studied, including in sentiment analysis, entity detection, conversational AI systems, etc. He et al. [31] proposed the DeBERTa model, which improves performance by enhancing the decoder and decomposing the attention mechanism. The initial component is the decomposed attention mechanism, which represents each word using two vectors encoding its content and position. The matrix’s decomposition of content and relative placements is used to calculate attention weights between words. Additionally, an improved mask decoder replaces the output softmax layer to predict mask tokens during model pre-training. Some researchers have proposed a model to integrate multi-modal information in biomedical named entity recognition tasks. These works continue to promote the development of the BERT model and achieve remarkable results in practical applications.

The extensive development of BERT in text processing provides significant technical support for tasks such as sensitive data identification and named entity recognition [32,33,34]. In the electric power industry, there exists a vast amount of data interaction between the State Grid business platform and third-party platforms, traversing numerous data flow paths. With high processing requirements and diverse data types, efficient processing is crucial. Leveraging BERT-based models can significantly enhance efficiency. 

### 2.3. Named Entity Recognition

Named Entity Recognition (NER) is a pivotal task in natural language processing(NLP), aiming to identify and classify named entities from text, such as names of people, places, organizations, etc. [35]. NER finds extensive application in information extraction, question-answering systems, machine translation, and various other fields.

Traditional NER methods, like rule-based approaches, incur substantial costs and heavily depend on manual steps such as rule construction and feature engineering. In recent years, some methods based on neural networks have gained traction in Named Entity Recognition tasks [36]. This approach leverages the LSTM-CRF framework, utilizing Long Short-Term Memory networks to capture implicit character representations and Conditional Random Fields for joint label decoding. Ma and Hovy also use the CNN to extract the feature representations of words [37]. First, they use the CNN to capture lexical features at the character level; then, the character-level representations and embedded words are concatenated and then fed into the RNN context encoder for processing. However, their model’s weaknesses include the absence of delimiters (spaces) and robust identifiers (capitalization), as well as inadequate training data, resulting in entity ambiguity. Guillaume et al. [38] employed BiLSTM to capture implicit text representations and utilized CRF for label decoding. On the other hand, Luo et al. [39] introduced a deep learning model incorporating a BiLSTM layer and a CRF layer (at-BiLSTM-CRF). They also attached an attention mechanism tailored for NER tasks in the chemical domain.

Compared with the Fully Connected Neural Network (FNN), which requires a fixed input length, the RNN is composed of cyclic units, can handle variable-length input data, and is more suitable for time-series inputs such as text data.

In addition, in order to not only focus on the feature extraction of words and the weights between words, but also ignore the semantic relationship of context, a large number of large language models have emerged. Since the emergence of large-scale pre-trained language models, research has indicated that incorporating these models as word-embedding layers can enhance the sequence labeling performance of models [40]. Mikolov et al. [41] introduced the word2vec model, a foundational context-free language model capable of converting words into dense vectors so that semantically similar words are closer in the vector space. Wang et al. [42] leveraged the BERT model in conjunction with the BiLSTM-CRF network, yielding favorable outcomes in Chinese resume entity recognition. Similarly, Yuan et al. [43] employed BERT and CNN models to extract text character and glyph feature vectors, subsequently fusing word vectors to extract text features across multiple dimensions. Their testing performance on the *People’s Daily* dataset surpassed that of other models.

In summary, the research methodologies concerning data leakage prevention, large-scale pre-training language models, and named entity recognition extend beyond those discussed in this section. The current widespread advancement of BERT in text processing offers substantial technical support for tasks involving sensitive data identification and named entity recognition. The electric power business domain encompasses a broad spectrum of data interactions between the State Grid business platform and third-party platforms, with various data flow paths. Given the high processing demands and diverse data types involved in large-scale data in the electric power sector, employing BERT-based models for data processing can significantly enhance efficiency. In Section 3, this article will detail the proposed framework for preventing power business data leakage, contextualized within the scenario of data interaction between the State Grid business platform and a third-party platform.

## 3. Data Leakage Prevention Framework Based on Deep Learning

### 3.1. Research Motivation

**Figure 1:**
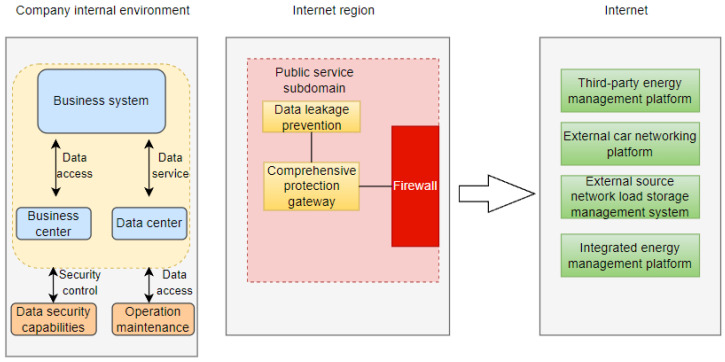
Data interaction framework diagram between State Grid business system and third-party platforms.

When the State Grid business system interacts with third-party platforms such as third-party energy management platforms, external Internet of Vehicles platforms, external source network load storage management systems, and integrated energy management platforms for power business data, it is necessary to interact through the integrated protection gateway of the public service subdomain of the Internet region. The third-party platform accesses the power grid business system through RESTful standard requests. The power grid business system returns normal business data to the third-party business and directs traffic to the data interaction and anti-leakage system through mirroring. The current data interaction situation between the State Grid business system and third-party platforms is shown in Figure 1.

Figure 1 shows the data interaction process between the State Grid business system and third-party platforms. A third-party platform may be attacked or illegally obtained and may send requests to obtain privacy-sensitive data. The State Grid business platform may return privacy-sensitive data to third-party platforms, causing power business data leakage. Power business data have the characteristics of long circulation paths and various types, as well as manual labeling problems caused by the large number of devices in power business scenarios, making manual feature selection complex.

In view of the above problems, this article proposes a deep learning-based power business data leakage prevention method. Therefore, data leakage prevention components are deployed on the comprehensive protection gateway to realize the sensitive content identification and filtering of externally released data. For the external interaction data of the power grid business in the public service domain, text content serves as the focal point for feature extraction. Utilizing detection rules such as regular expressions and keywords, we construct a sensitive data distribution library from sampled data. In power grid business systems, privacy-sensitive information encompasses identity cards, phone numbers, email addresses, and other information.

The traffic data is divided according to fields, and the data is preprocessed by cutting, conversion, and cleaning. This includes merging and deduplicating text content, and ultimately generating sample sets.

### 3.2. Privacy-Sensitive Data Identification Based on Regular Expressions

Determine the fixed structure of sensitive data, $R_{U_{i}}^{1}=r_{U_{i}}^{1},r_{U_{i}}^{2},\ldots .,r_{U_{i}}^{R}$, for each specific user $u_{i}$. For each rule, $r_{U_{i}}^{k}$ is a privacy rule with a specific structure associated with the user.

(1) $$r_{i}\leftarrow f_{1}\wedge f_{2}\wedge \ldots \wedge f_{L}$$

where $r_{i}$ represents the target-sensitive rule, and each $f_{k}$ is represented as a logical expression of an instance attribute. L mainly represents the length of the rule.

For structured power business sensitive data interacting between the State Grid business platform and third-party platforms, regular expressions can be employed for matching. Structured data typically consist of ID numbers, phone numbers, email addresses, etc., and are composed of alphanumeric characters. Such data types can be directly matched using regular expressions.

For structured data, use regular expressions for matching. Structured data include ID numbers, phone numbers, email addresses, IP addresses, etc. 

Use the regular expression/^[a-z]([a-z0-9]*[-_]?[a-z0-9]+)*@([a-z0-9]*[-_]?[a-z0-9]+)+[\.][a-z]{2,3}([\.][a-z]{2})?$/i can detect email addresses.

[a-z] means the first character of the username must be a lowercase letter from a to z. ([a-z0-9]*[-_]?[a-z0-9]+)* is used to represent the username part. [a-z0-9]* represents a character class that includes all lowercase letters (a to z) and digits (0 to 9). The asterisk (*) means that this character class can appear zero or more times. [-_]? indicates that there can be zero or one hyphen (-) or underscore (_).

[a-z0-9]+ indicates that there must be one or more lowercase letters or numbers. The plus sign [...]+ means that this character class must appear at least once. The combination part (...)* indicates that the above pattern can be repeated zero or more times. 

([a-z0-9]*[-_]?[a-z0-9]+)+ is used to represent the domain name part. The rules for this part are similar to the rules for the username. (...)+ indicates that the above pattern must appear at least once. 

[\.][a-z]{2,3} indicates that the top-level domain part, such as ‘.com’. [\.] represents the dot (.). [a-z]{2,3} means that the preceding character class [a-z] should match at least two times and at most three times. 

([\.][a-z]{2})? indicates an optional second-level top-level domain, such as ‘.co.uk’. The specific meaning of the characters is similar to the above.

For example, for ‘john.doe@gmail.com’, ‘john.doe’ matches the username part [a-z]([a-z0-9]*[-_]?[a-z0-9]+)*, ‘gmail’ matches the domain part ([a-z0-9]*[-_]?[a-z0-9]+)+, and ‘.com’ matches the top-level domain part [\.][a-z]{2,3}.

Use the regular expression ^[1-9]\d{5}[1-9]\d{3}((0\d)|(1[0-2]))(([0|1|2]\d)|3[0-1])\d{4}[0-9Xx] to detect an ID number. 

[1-9]\d{5} matches the area code of the ID number, and [1-9] matches any single digit from 1 to 9. It does not match 0. ‘\d’ matches any digit from 0 to 9; {5} is a quantifier that specifies exactly five occurrences of the preceding element; and [1-9]\d{3} matches the year of birth in the ID number. 

((0\d)|(1[0-2])) ensures the validity of the month. ‘(0\d)’ matches ’01’ to ‘09’ and (1[0-2]) matches ‘10’ to ‘12’; (([0|1|2]\d)|3[0-1]) indicates the date, matching ‘01’ to ‘31’. \d{4} matches the sequence code in the ID number. \d{4} specifies exactly four occurrences of the preceding element, which in this case is a digit (\d). [0-9Xx] indicates that the last digit can be a number 0–9 or a letter X/x (check digit).

Use the regular expression ((d{11})|^((d{7,8})|(d{4}|d{3})-(d{7,8})|(d{4}|d{3})-(d{7,8})-(d{4}|d{3}|d{2}|d{1})|(d{7,8})-(d{4 }|d{3}|d{2}|d{1}))$) to detect a phone number. 

(\d{11}) indicates 11 consecutive digits, usually a normal mobile phone number. The vertical bar ‘|’ is an alternation operator that acts like a logical OR. (\d{7,8}) matches a local number with seven to eight digits. 

(\d{4}|\d{3})-(\d{7,8}) matches a phone number with an area code. (\d{4}|\d{3}) indicates the area code, which can be three or four digits. 

(\d{4}|\d{3})-(\d{7,8})-(\d{4}|\d{3}|\d{2}|\d{1}) matches a phone number with an area code and an extension. (\d{4}|\d{3}|\d{2}|\d{1}) indicates the extension, which can be one to four digits. 

(\d{7,8})-(\d{4}|\d{3}|\d{2}|\d{1}) matches a local number with an extension. For example, ‘18812341354’ matches an 11-digit mobile phone number, and ‘010-12345678’ matches a local number with a three-digit area code.

An IPv4 address usually consists of four groups of numbers, each ranging from 0 to 255. Use the regular expression ((?:[0,1]?\d{1,2}|2(?:[0-4][0-9]|5[0-5]))(? :\ .(?:[0,1]?\d{1,2}|2(?:[0-4][0-9] |5[0-6]))) +{3})+ to detect an IP address. 

(?:[0-1]?\d{1,2}|2(?:[0-4]\d|5[0-5])) is used to match values from 0 to 255. (?: ... ) represents non-capturing groups that are used to group parts of the pattern without capturing the matched text for back-referencing, and [0-1]? matches zero or one occurrence of the digits 0 or 1. \d{1,2} matches one or two digits (0-9). [0-1]?\d{1,2} matches numbers from 0 to 199, and 2(?:[0-4][0-9]|5[0-5]) covers numbers from 200 to 255.

‘\.’ matches the period. (?:[0-1]?\d{1,2}|2(?:[0-4]\d|5[0-5])) repeats the logic of the first octet and matches the value from 0 to 255, while {3} means repeating the previous pattern three times, i.e., the first three values. For example, the four groups of numbers in ‘192.168.0.1’ are all in the range of 0 to 255.

These data adhere to fixed format requirements, making detection and identification relatively straightforward. However, when dealing with a large volume of sensitive data requiring prevention from leakage, more advanced semantic analysis and machine learning technologies become essential for accurate identification.

### 3.3. Privacy-Sensitive Data Identification Based on DeBERTa-BiLSTM-CRF

#### 3.3.1. DeBERTa Layer

Unstructured sensitive data usually have a flexible format with no fixed format requirements. These data may be in documents or images. The identification of these data requires semantic understanding and context analysis, and based on the context, it is determined whether the data contain sensitive information that needs to be prevented from being leaked. The electric power business has many types of data structures and a large amount of data. There are correlations between different data types. However, the huge amount of data is difficult to monitor manually.

**Figure 2:**
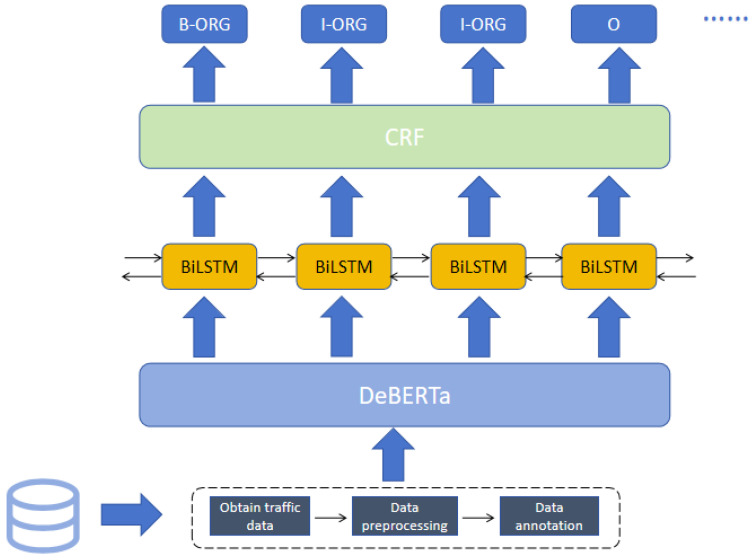
Model structure.

For detecting leak-proof private data within these datasets, the DeBERTa-BiLSTM-CRF model is utilized. The model architecture is shown in Figure 2.

A sequence of Transformer blocks with a self-attention mechanism layered on top of each other make up the BERT architecture [44]. The formula for calculating the attention score is expressed as follows:

(2) $$Attention(Q,K,V)=Softmax\left(\frac{QK^{T}}{\sqrt{d_{k}}}\right)V$$

Among them, Q,K, and V are all word-embedding representations. $W=[W^{Q},W^{K},W^{V}]$ represents the weight matrix of the multi-head attention mechanism. Each transformer block takes word embeddings, which are constructed through the encoding of word vectors, as the input.

During the utilization of BERT, two main steps are involved: pre-training and fine-tuning. In pre-training, the model undergoes unsupervised training across various tasks. Subsequently, in fine-tuning, the model is initialized with pre-trained parameters and further refined for specific tasks under supervision. Leveraging the pre-trained DeBERTa model, training and fine-tuning operations are performed on the power business dataset to align it more closely with the scenario of power business data recognition.

The DeBERTa model uses a decoupled attention mechanism and enhanced mask decoder to improve BERT model. In the original BERT model, each word or character is represented by a vector. In this model, each word is represented by two vectors, one encoding content and one encoding position, respectively, and the attention weights among words are computed using disentangled matrices on theirs content and relative position. When calculating the attention weight, content-related calculations use the content matrix, and position-related calculations use the position matrix, resulting in the decoupling of content and position.

$\left\{H_{i}\right\}$ represents the content vector and $\left\{P_{i|j}\right\}$ represents the relative position vector of position i relative to position j in the sequence. The attention weight of the word pair (i, j) can be calculated by using the decoupled matrix of content and position, as shown in Equation (3).

(3) $$\begin{array}{cc} A_{i,j} & =\{H_{i},P_{i|j}\}\times \{H_{j},P_{j|i}{\}}^{T}\\ & =H_{i}H_{j}^{T}+H_{i}P_{j|i}^{T}+P_{i|j}H_{j}^{T}+P_{i|j}P_{j|i}^{T} \end{array}$$

Masked Language Modeling (MLM) is used in the pre-training of the DeBERTa model, akin to the BERT model. In this process, the model learns to predict masked words using surrounding context. However, DeBERTa enhances MLM by incorporating both content and positional information from the context. Although the decoupled attention mechanism in DeBERTa considers content and relative position, it overlooks the absolute position of words, which is often pivotal for accurate prediction. Many grammatical subtleties rely heavily on the absolute position of words within a sentence.

There are two approaches to incorporate absolute positions. The BERT model includes absolute positions in the input layer. On the other hand, in DeBERTa, absolute positions are merged across all Transformer layers, with mask token prediction conducted before the softmax layer, as depicted in Figure 2. This design enables DeBERTa to capture relative positions across all Transformer layers while utilizing absolute positions as additional information during masked word decoding. This mechanism is referred to as DeBERTa enhanced mask decoder (EMD).

Due to the numerous circulation paths of power business data, the high processing demands for large-scale data, and the diverse range of data types, power business data typically necessitate relatively standardized formatting requirements, with variations across different data types. Consequently, textual sequences require character-level representation. Beyond absolute positions, the model also needs to consider relative positions between characters to accurately capture dependencies between words, encompassing their content and relative positions. Moreover, the DeBERTa model excels at capturing long-distance dependencies between words and outperforms the RoBERTa model on extended sequences, thus addressing the challenges posed by lengthy data sequences in the power business domain and enhancing recognition and matching capabilities.

#### 3.3.2. BiLSTM Layer

Following the BERT model, a Bidirectional Long Short-Term Memory Network (BiLSTM) is introduced. BiLSTM is adept at capturing contextual information within a sequence by simultaneously considering both preceding and succeeding words. It is composed of forward and backward LSTMs, whose outputs are concatenated after processing. BiLSTM is commonly employed in NLP tasks for modeling context.

Effective information is preserved and selected through processes of forgetting and remembering within Long Short-Term Memory (LSTM). At each time step, forgetting, remembering, and output are regulated by forget gates, memory gates, and output gates, respectively. These gates are computed based on the hidden state from the previous time step and the current input. 

The principle and calculation processes of a certain unit at a certain moment within the BiLSTM structure are as follows.

Step 1: Calculate the forgetting gate to decide what information to forget or discard from the unit state. Obtain both the current and the concealed state $h_{t-1}$ from the previous instant. The sigmoid function σ uses the preceding time $x_{t}$ as input, and outputs a value $f_{t}$ between (0,1) to show the degree of forgetting the knowledge in the unit state $C_{t-1}$ (0: totally forgotten; 1: completely accepted). The calculation formula is

(4) $$f_{t}=\sigma \cdot (W_{f}\cdot [h_{t-1},x_{t}]+b_{f})$$

In the formula, $b_{f}$ is the forget gate bias vector.

Step 2: To decide what new information to keep from the cell state, compute the input gate. (1) Take the input of the hidden state $h_{t-1}$ from the previous moment and the current moment $X_{t}$. (2) Use the tanh layer to create a new candidate vector ${\tilde{C}}_{t}$, which is added to the unit state. (3) Calculate and output a value $i_{t}$ between (0,1) to indicate which information in the unit state $C_{t-1}$ needs to be updated. The calculation formula is

(5) $$i_{t}=\sigma \cdot (W_{i}\cdot [h_{t-1},x_{t}]+b_{i})$$

(6) $${\tilde{C}}_{t}=tanh\cdot (W_{c}\cdot [h_{t-1},x_{t}]+b_{c})$$

where $b_{c}$ is the bias vector of the memory unit and $b_{i}$ is the bias vector of the update gate.

Step 3: Update the unit state $C_{t-1}$ at the previous moment to the unit state $C_{t}$ at the current moment. The calculation formula is

(7) $$C_{t}=f_{t}\cdot C_{t-1}+i_{t}\cdot {\tilde{C}}_{t}$$

Step 4: Determine what information needs to be output by computing the output gate and the hidden state $h_{t}$ at the present time. Select which information from the unit state $C_{t-1}$ needs to be produced by receiving the input of the hidden state $h_{t-1}$ at the previous moment and the present moment $X_{t}$. Then, input the unit state $C_{t}$ into the tanh layer for processing, finally perform a product operation with $o_{t}$ to output the information we need. The calculation formula is

(8) $$o_{t}=\sigma \cdot (W_{o}\cdot [h_{t\cdot 1},x_{t}]+b_{o})$$

(9) $$h_{t}=\sigma \cdot tanh(C_{t})$$

where $b_{o}$ is the output gate bias vector.

The BiLSTM layer can extract sequence context semantic features and input the features to the CRF layer.

#### 3.3.3. CRF Layer

After the BiLSTM layer, a Conditional Random Field (CRF) layer is added [45]. The CRF layers dependencies between tags across the entire annotation sequence, ensuring the consistency of the generated annotation sequences.

CRF is a probabilistic graphical model used to solve sequence labeling problems. It receives an observation sequence ($X_{1}$, $X_{2}$, …, $X_{n}$) and outputs a state sequence ($Y_{1}$, $Y_{2}$, …, $Y_{n}$). The calculation method is to calculate the corresponding score of the sentence label through the emission score and transition score output by BiLSTM. The calculation formula is

(10) $$score(x,y)=\sum_{i=1}^{n}P_{i,y_{i}}+\sum_{i=1}^{n}A_{y_{i},y_{i+1}}$$

where $P_{i,y_{i}}$ is the score of the i-th character predicted to be the $y_{i}$ label; $A_{y_{i},y_{i+1}}$ is the score of the $y_{i}$ label transferred to the label $y_{i+1}$.

Consider a sentence with n words, each having m possible tags. This yields ${m\;}^{n}$ possible tag sequences for the sentence. The CRF layer assigns a score to each possible label sequence by learning the adjacent dependencies between label sequences. The sequence with the highest score is identified as the optimal label sequence, determining the category of the named entity. For instance, in the sequence labeling task, the first word in a sequence is typically label as “B-” or “O-”, and cannot be labeled as “I-” according to certain rules. By adhering to these rules, the CRF layer outputs the optimal label sequence.

### 3.4. Response Handling

If the model identifies certain data as sensitive information within the electric power business, we will desensitize any privacy-sensitive data it contains. Concurrently, we will issue real-time alerts and implement interception measures for data flows containing sensitive information to safeguard data security. The original data will be retained for risk analysis, enabling us to develop a method for preventing leakage in power business data interaction.

The power business data interactive leakage prevention system encompasses abnormal traffic detection, which involves establishing a traffic-monitoring system to conduct the real-time monitoring of data traffic and access patterns. This monitoring includes tracking data sources, destinations, frequencies, and other relevant metrics. Anomaly detection algorithms, such as statistical methods, machine learning, or deep learning techniques, are employed to identify irregular behaviors like large-scale data downloads or abnormal access frequencies.

For post-event response, an alarm system is established to promptly detect and notify the security team or administrator of abnormal events. Additionally, a comprehensive emergency response plan is formulated, outlining processing procedures, responsible personnel, communication channels, etc., to address security incidents promptly. Data are backed up regularly, and a data-recovery mechanism is established to mitigate potential data leakage or damage.

Furthermore, investigations and audits are conducted following security incidents to analyze their causes and impacts. Corresponding measures are then implemented to reinforce security protection based on the findings of these investigations.

The specific process of this electric power business data interaction and leakage prevention method is shown in Algorithm 1.
**Algorithm 1** Method for preventing leakage of electric power business data interactionInput: electric power business data Output: sensitive information of electric power business data1:Divide traffic data according to fields and perform preprocessing operations on the data2:Determine $R_{U_{i}}^{1}=r_{U_{i}}^{1},r_{U_{i}}^{2},\ldots .,r_{U_{i}}^{R}$ for each specific user $u_{i}$
3:Use regular expressions rule $R_{U_{i}}$ to match structured power business sensitive data4:Use the pre-trained DeBERTa model to extract text feature vectors F5:$\begin{array}{cc} A_{i,j} & =\{H_{i},P_{i|j}\}\times \{H_{j},P_{j|i}{\}}^{T}\\ & =H_{i}H_{j}^{T}+H_{i}P_{j|i}^{T}+P_{i|j}H_{j}^{T}+P_{i|j}P_{j|i}^{T} \end{array}$6:Calculate isentangled Attention:7:**for** i=0,…,N **do**8:$\;\;\;\;\;\;{\tilde{A}}_{c\to p}\left[i,:\right]=Q_{c}\left[i,:\right]K_{r}^{T}$9:**end for**10:**for** i=0,…,N−1 **do**11:      **for** j=0,…,N−1 **do**12:            
$A_{c\to p}[i,j]={\tilde{A}}_{c\to p}[i,\delta [i,j]]$
13:      
**end for**
14:**end for**15:**for** j=0,…,N−1 do16:$\;\;\;\;\;\;\;\;{\tilde{A}}_{p\to c}[:,j]=K_{c}[j,:]Q_{r}^{T}$17:**end for**18:**for** j=0,…,N−1 do19:      **for** i=0,…,N−1 do20:            
$A_{p\to c}[i,j]={\tilde{A}}_{p\to c}[\delta [j,i],j]$
21:      
**end for**
22:**end for**23:$\tilde{A}=A_{c\to c}+A_{c\to p}+A_{p\to c}$24:Use BiLSTM model to capture contextual information in input sequences S25:$h_{i}=BiLSTM(x_{i})$26:$score(x,y)=\sum_{i=1}^{n}P_{i,y_{i}}+\sum_{i=1}^{n}A_{y_{i},y_{i+1}}$27:Carry out alarm and blocking operations for sensitive power business data 

## 4. Experiment

### 4.1. Experiment Settings

In order to verify the effectiveness of the anti-leakage method for power data interaction between the State Grid business platform and third-party platforms, the sensitive data identification method of the data leakage prevention system was verified using a dataset. The matching of leak-proof sensitive data was analogized to named entity recognition [27]. In the experiments of this article, some categories in the entity category are used as categories of leak-proof data, and the identification and matching operations are performed on the data belonging to this category.

As the data exchanged between the State Grid business platform and third-party platforms in the electric power industry are considered confidential within the company, direct access to this data is restricted. Therefore, in this experiment, the CLUENER 2020 dataset was utilized. The CLUENER 2020 dataset is designed for named entity recognition, and comprises training, verification, and test sets. It encompasses ten label categories, namely: address, book, company, game, government, movie, name, organization, position, and scene. The distribution of entity categories in the CLUENER 2020 dataset is shown in Table 1. In the experiment, within the CLUENER 2020 dataset, certain named entities (such as person names, addresses, and organization names) are identified as privacy sensitive data, simulating the identification of sensitive data to prevent leakage during data interactions between the State Grid business platform and third-party platforms. In this paper, the BIO tagging strategy is adopted, that is, the first character of a named entity is tagged with the “B” tag, subsequent characters within the same entity are tagged with the “I” tag, and all other non-named entities are tagged with the “O” tag. 

This article conducts experiments in the environment shown in Table 2.

This DeBERTa model consists of 24 layers, 16 attention heads, and 1024 hidden layer dimensions. During training, the batch size is 32, and the learning rate is fixed at 0.00003. The AdamW optimizer, a variant of the Adam optimizer that incorporates modifications to the weight decay for better regularization control, is utilized for model optimization. To mitigate overfitting, dropout regularization is applied, with a dropout rate of 0.3 for the DeBERTa layers. Furthermore, the BiLSTM component of the model has a hidden layer size of 256. 

The hyperparameters of this experiment are shown in Table 3.

### 4.2. Evaluation Metrics

The metrics used to evaluate named entity recognition are precision *P*, recall *R*, and F1-score. Precision is defined as the ratio of correctly recognized named entities to the total number of recognized named entities, and recall represents the ratio of recognized named entities to the total number of named entities contained in the text. F1 is a comprehensive measure of precision and recall. It is the harmonic mean of model precision and recall. Theirs formulas are as follows:

(11) $$P=\frac{T_{P}}{T_{P}+F_{P}}\times 100\%$$

(12) $$R=\frac{T_{P}}{T_{P}+F_{n}}\times 100\%$$

(13) $$F1=2\times \frac{P\times R}{P+R}\times 100\%$$

$T_{P}$ represents the count of correctly identified entities by the model, $F_{P}$ indicates the count of irrelevant entities identified by the model, and $F_{n}$ denotes the number of relevant entities not detected by the model.

In addition to F1-score, $F_{0.5}$-score and $F_{2}$-score are also widely used in statistics. Theirs formulas are as follows:

(14) $$F_{\beta }=\left(1+\beta^{2}\right)\times \frac{P\times R}{\beta^{2}\times P+R}$$

(15) $$F_{0.5}=\frac{5}{4}\times \frac{P\times R}{\frac{P}{4}+R}$$

(16) $$F_{2}=5\times \frac{P\times R}{4\times P+R}$$

In the $F_{0.5}$-score, the β value in Formula (14) is set to 0.5, and the weight of precision is higher than recall. In the $F_{2}$-score, the β value in Formula (14) is set to 2, and the weight of recall is higher than precision.

Here, we compare our scheme with the following methods:

BiLSTM-CRF: The BiLSTM network can capture forward information and reverse information at the same time, making more comprehensive use of text information, and it uses the CRF layer to obtain the globally optimal label sequence.

BERT: BERT is a pre-trained language model built on the Transformer architecture. It learns rich language representations through large-scale unsupervised language model pre-training tasks.

BERT-BiLSTM-CRF: Combining the BERT model with BiLSTM-CRF, this model uses the BERT model’s semantic understanding of text, the BiLSTM model’s ability to capture sequence information, and the CRF model’s modeling of the transition probability between tags.

RoBERTa (Robustly optimized BERT approach) is a pre-trained language representation model based on BERT improvement. RoBERTa adopts a more flexible masking strategy that masks parts of words in the input text and requires the model to predict the positions of these masks, instead of randomly masking 50% of the words like BERT.

### 4.3. Experimental Results and Analysis

In order to verify the effectiveness of the method in this article, the method in this article is compared with regular expressions, as well as some baseline methods of named entity recognition, i.e., the BiLSTM-CRF, BERT, BERT-BiLSTM-CRF, and RoBERTa [28] models, with precision rate, recall rate, and F1 score used as indicators for model evaluation. 

As can be seen from Table 4, the precision, recall rate and F1-score of the DeBERTa-BiLSTM-CRF model have been improved to a certain extent. The comparative experimental results show that the recognition ability of sensitive data has been improved to a certain extent compared with the common BERT-BiLSTM-CRF model, and it has better generalization ability than regular expression matching. 

Our model outperforms BiLSTM-CRF, BERT, and RoBERTa in terms of precision, recall, and F1-score. Its F1-score is 81.26%, an improvement of 0.84% over the RoBERT model. It achieves 80.13% and 82.41% in precision and recall, an improvement of 0.87% and 0.72% over the RoBERT model. The DeBERTa-BiLSTM-CRF model also has a higher $F_{0.5}$-score and $F_{2}$-score of 80.58% and 81.94%, respectively, an improvement of 0.85% and 0.75% over the other methods. The $F_{0.5}$-score places more emphasis on precision, and the $F_{2}$-score places more emphasis on recall. Our method can improve both indicators.

**Figure 3:**
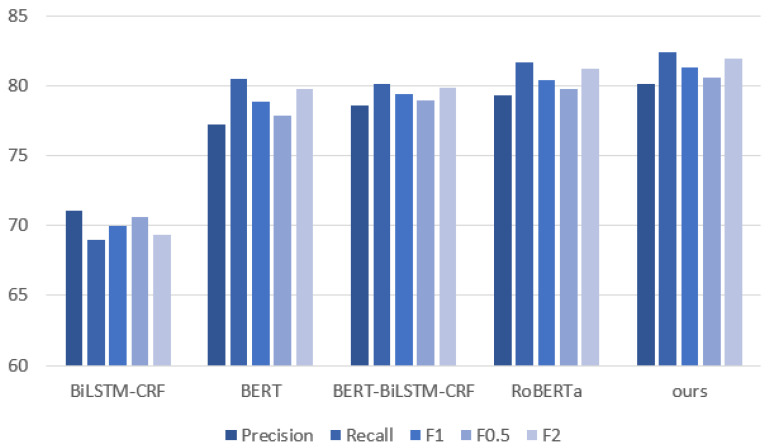
Precision, recall, F1-score, $F_{0.5}$-score, and $F_{2}$-score of different models.

Additionally, to provide a more intuitive visualization of the experimental data, a histogram is utilized to present and compare the results of different models in Figure 3. It is evident that the model based on BERT substantially enhanced the F1-score compared to the traditional BiLSTM-CRF model, increasing this value from 70.00% to about 80%. This demonstrates the effectiveness of applying the BERT model to the named entity recognition task.

BiLSTM-CRF has limited feature representation capabilities and weak processing capabilities for polysemy and long-distance dependencies, resulting in inaccurate results. In addition, although BERT and RoBERTa consider contextual information, they ignore the dependencies between words. This makes their performance better than BiLSTM-CRF, but worse than the model proposed in this paper. Furthermore, although BERT and RoBERTa consider contextual information, they ignore absolute position information. This makes them perform better than LSTM-CRF, but worse than the model proposed in this paper. Our method uses the DeBERTa model. DeBERTa has improved performance compared to the BERT and RoBERTa models, using a decoupled attention mechanism and introducing absolute position coding. It significantly improved pre-training efficiency and downstream task performance. 

**Figure 4:**
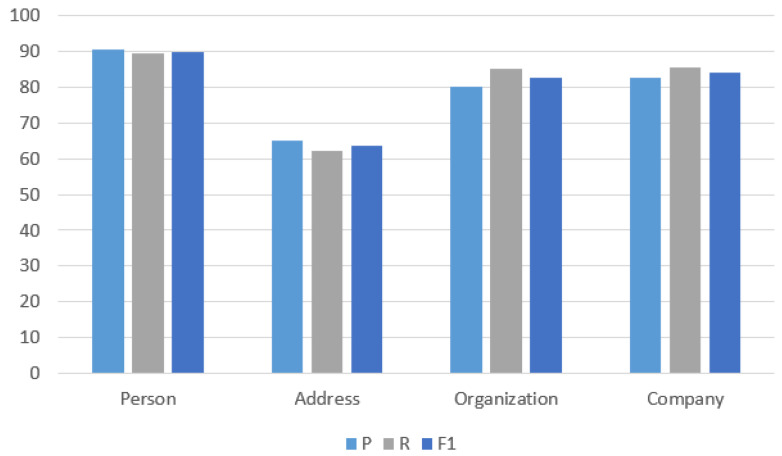
Precision, recall, and F1-score of entities belonging to sensitive data.

Figure 4 shows the precision rate, recall rate, and F1-score of the identified entity categories of person, address, organization, and company, which are marked as sensitive data entities. It can be seen that good results can be achieved for the entity categories of person, organization, and company. Specifically, the precision, recall, and F1-score for the person category achieve 90.46%, 89.33%, and 89.89%, respectively. However, the F1 score for the address category stands at only 63.54%, indicating a need for further optimization in recognition effectiveness. This discrepancy may arise from the diverse forms and formats typically associated with addresses. Address expressions can vary across regions and cultural backgrounds, often necessitating reliance on contextual information for accurate identification. Insufficient or irrelevant contextual cues pertaining to address entities can impede accurate identification.

**Figure 5:**
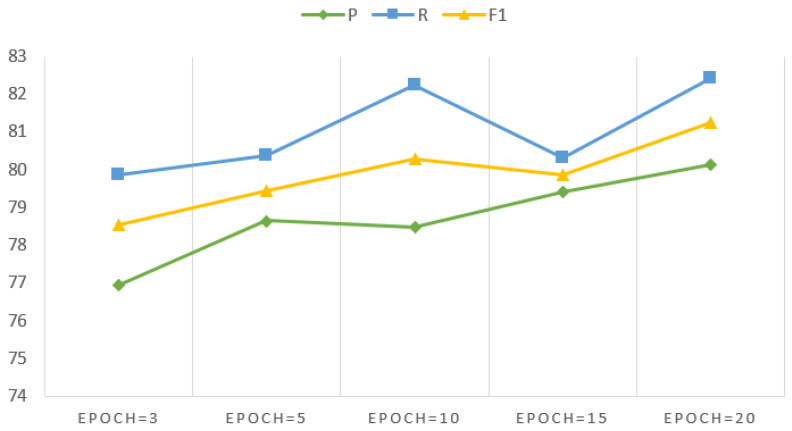
Precision, recall and F1 score of different epochs.

Figure 5 illustrates the precision, recall, and F1-score values across 3, 5, 10, 15, and 20 training epochs, respectively. As can be seen in the figure, when the number of training epochs ranges from 3 to 10, the F1-score demonstrates a gradual increase, rising from 78.52% to 80.28%. However, with 15 training epochs, the F1-score experiences a decline. It is evident that both the F1-score and the recall value exhibit an initial increase, followed by a decrease. The most favorable outcomes were observed in the 20 epochs regarding F1-score. Nonetheless, the enhancement relative to the 10 training epochs is modest.

To analyze the recognition effect of different sensitive entity categories, the DeBERTa-BiLSTM-CRF method is compared with the commonly used named entity methods for the person, address, organization, and company categories defined as sensitive entity categories. The F1-score results are shown in Table 5.

As can be seen from Table 5, among the four defined sensitive entity categories, the F1 score can be improved compared to the baseline method. In particular, the F1-score of the person category can reach 89.89%. For the recognition of the address entity, compared with the traditional named entity recognition model, the F1-score of the BiLSTM-CRF model is only 45.50%, while the F1-score of our method is 63.54%, which is a significant improvement.

As can be seen from Table 6 and Table 7, the $F_{0.5}$-score improved compared to the RoBERTa model. In particular, the recognition success rate of address sensitive entities increased by 1.40%. The $F_{2}$-score of person entities and organization decreased to a small extent. Since the $F_{2}$-score focuses more on recall ratio, the partial decrease in recall rate will affect the final $F_{2}$-score.

From the perspective of algorithmic complexity, BiLSTM-CRF has a lower algorithmic complexity, but its performance is limited when dealing with long-distance dependencies. BERT effectively captures long-distance dependencies through its self-attention mechanism, improving representation capabilities. However, its Transformer structure brings higher computational complexity, especially when dealing with long sequences. As an improved version of BERT, RoBERTa improves performance with larger data volumes and longer training times, but also increases computational costs accordingly. DeBERTa introduces relative position encoding and mask decoder mechanisms based on BERT, further enhancing the model’s ability to capture complex language phenomena. After being combined with BiLSTM-CRF, although the complexity increases, the model can handle named entity recognition tasks more effectively.

From the perspective of computational load, BiLSTM-CRF has a lower computational load and is suitable for resource-constrained environments. BERT and RoBERTa have higher computational loads and require powerful hardware support, especially in a multi-layer Transformer architecture. BERT-BiLSTM-CRF and DeBERTa-BiLSTM-CRF have higher computational loads, but DeBERTa’s relative position encoding and mask decoder mechanism further optimize the model architecture, and the model performs better with the same computing resources. At the same time, DeBERTa removes the Next Sentence Prediction (NSP) task in the pre-training of BERT, and the optimization of the pre-training strategy further reduces DeBERTa’s resource requirements in the pre-training stage.

In terms of characteristics, the BiLSTM-CRF model has a small computational load and fewer model parameters, but its performance is relatively average. BERT is a powerful pre-trained model with good generalization ability. RoBERTa requires a large amount of data for pre-training and has a longer training time than BERT. BERT-BiLSTM-CRF combines the representation ability of BERT with the sequence labeling ability of BiLSTM-CRF. In the Chinese NER task, character-level information is very important. DeBERTa was designed with full consideration of the characteristics of the Chinese language. It is more sophisticated in processing character-level representation and contextual understanding. A large amount of Chinese corpus was used in pre-training, which further improved the performance of the model in Chinese tasks.

Based on the experimental results and the analysis presented above, our proposed model demonstrates better performance compared to the BiLSTM-CRF, BERT, BERT-BiLSTM-CRF, and RoBERTa models. Additionally, it exhibits greater generalizability than regular expressions. Notably, among the four defined sensitive entity categories, our method excels particularly in recognizing privacy-sensitive data. In summary, this approach effectively mitigates the risk of power business data leakage, thus furnishing a dependable safeguard for the security of power business data interactions.

## 5. Conclusions

In this paper, we propose a deep learning-based anti-leakage method for power business data interactions. When this method interacts with power business data between the State Grid business platform and a third-party platform, it is necessary to match the privacy-sensitive data of the interactive power business data to prevent the leakage of data to external platforms. We use a combination of regular expressions and an improved BERT models to obtain good matching results on structured and unstructured data, thereby identifying power-sensitive data and desensitizing privacy-sensitive information in interaction. Sensitive information traffic is alerted and intercepted online, and the original data are retained to build an anti-leakage method for power business data interaction. Finally, the experimental results reaffirm the feasibility and effectiveness of the method proposed in this paper for ensuring the data security of the Power Industry Internet of Things compared with representative baseline methods.

However, our method still exhibits some limitations. For instance, we necessitate pre-defining the data entity categories that fall under the sensitive data category. This poses a challenge when dealing with data that we wish to classify as sensitive but do not align with any predefined category. In such cases, there is a lack of effective identification strategies. Hence, enhancing the diversity of recognized entity types emerges as a focal point of our future research endeavors to bolster our approach. Additionally, we contemplate incorporating data preprocessing enhancement strategies to refine the recognition effectiveness further.

## Figures and Tables

**Table 1:** Entity distribution in the CLUENER 2020 dataset.

Entity Class	Train	Dev
Address	2829	364
Book	1131	152
Company	2897	366
Game	2325	287
Government	1797	244
Movie	1109	150
Name	3661	451
Organization	3075	344
Position	3052	425
Scene	1462	199

**Table 2:** Experiment environment.

CPU	Intel(R) Xeon(R) Gold 6230 CPU @ 2.10 GHz
RAM	64G
GPU	NVIDIA GeForce RTX 3090
Compiler environment	python 3.7+ pytorch 1.9
Operating system	Linux 3.10.0-1160

**Table 3:** Hyperparameters.

Learning rate	0.00003
Attention heads	16
Hidden size	1024
Batch size	32
Dropout rate	0.3
Maximum sequence length	128
BiLSTM hidden layer	256

**Table 4:** Precision, recall, F1 score, $F_{0.5}$ score, and $F_{2}$ score for different models.

Model	Precision	Recall	F1-Score	$F_{0.5}$-Score	$F_{2}$-Score
Regular expression	100	\	\	\	\
BiLSTM-CRF	71.06	68.97	70.00	70.63	69.38
BERT	77.24	80.46	78.82	77.86	79.79
BERT-BiLSTM-CRF	78.62	80.15	79.38	78.92	79.84
RoBERTa	79.26	81.69	80.42	79.73	81.19
Ours	80.13	82.41	81.26	80.58	81.94

**Table 5:** F1-score of different models for sensitive entity categories.

	BiLSTM-CRF	BERT	RoBERTa	Ours
Person	74.04	88.75	89.09	89.89
Address	45.50	60.89	62.63	63.54
Organization	75.96	79.43	82.34	82.56
Company	72.27	81.42	83.02	84.10

**Table 6:** $F_{0.5}$-score of different models for sensitive entity categories.

	BiLSTM-CRF	BERT	RoBERTa	Ours
Person	76.40	88.26	88.44	90.23
Address	48.10	59.56	63.01	64.41
Organization	74.79	76.64	80.51	80.99
Company	73.97	81.35	82.71	83.33

**Table 7:** $F_{2}$-scores of different models for sensitive entity categories.

	BiLSTM-CRF	BERT	RoBERTa	Ours
Person	71.83	89.25	89.76	89.55
Address	43.17	62.28	62.25	62.69
Organization	77.15	82.43	84.25	84.21
Company	70.66	81.48	83.33	84.88

## Data Availability

Data are contained within the article.
